# Manna to Gall

**DOI:** 10.3201/eid1701.AC1701

**Published:** 2011-01

**Authors:** Polyxeni Potter

**Affiliations:** Author affiliation: Centers for Disease Control and Prevention, Atlanta, Georgia, USA

**Keywords:** Art science connection, emerging infectious diseases, art and medicine, Peter Paul Rubens, The Gathering of the Manna, foodborne infections, Manna to Gall, Baroque painting, about the cover

“No undertaking, however vast, has ever surpassed my courage,” professed Peter Paul Rubens, whose work comprised cathedral domes and altarpieces, portraits, landscapes, and designs for sculpture and architecture. Courage also drove his exuberant style of flamboyant color and movement. “That Homer of painting, the father of warmth and enthusiasm in art,” said of him by Eugene Delacroix, eclipsed most other artists, “Not because of his perfection in any one direction, but because of that hidden force―that life and spirit―which he put into everything he did.” Delacroix, who borrowed and absorbed much from him, wrote in his personal journal that his admiration was so great, he “cared to be Rubens.”

He was described as of “tall stature, a stately bearing … rosy cheeks, chestnut brown hair, sparkling eyes but with passion restrained, a laughing air, gentle and courteous.” Known as “the Apelles of his age” and “the most learned painter in the world,” Rubens was hardworking; multilingual; a prodigious collector; and a fine diplomat, negotiating treaties all over Europe for more than 25 years. His friend the theologian Gaspar Scioppius wrote, “I know not what to praise most, his ability in painting, in which he attains the most exalted rank attained by any man of his century, or his knowledge of literature, his enlightened taste, and the all too rare agreement between his words and his deeds.”

Rubens’ career spanned a tumultuous age in Europe, filled with political, religious, and cultural developments, many of which he was able to influence. Shifting geographic borders and denominational allegiances often worked to his advantage as his diplomatic connections brought him royal commissions and generous court assignments. An age of contradictions, the 17th century saw in the sciences a move toward specific methods of inquiry, while the arts turned to an imaginative style. Bourgeois capitalism was on the rise, and the powerful of Europe were building exquisite palaces. Italian art was spreading everywhere, while Italy was losing its edge in international trade. This, known as the age of the Baroque, produced many great masters, among them Caravaggio; Bernini; and Rubens, who epitomized and defined it.

A native of Siegen, Germany, Rubens lived and went to school in Antwerp, where he became a master. According to his nephew, he was soon “seized with a desire to see Italy and to view at first hand the most celebrated works of art, ancient and modern, in that country, and to form his art after these models.” In Venice, he studied Titian, Veronese, Bassano, and Tintoretto, whose influences guided “the great speed and furor of his brush.” His following so grew that soon he would call himself “the busiest and most harassed man in the world.”

When he returned to Antwerp, his large studio employed some of the greatest Flemish masters of the day, among them Anthony van Dyck and Frans Snyders. He relied on their work to meet demand, something he openly acknowledged. Danish physician Otto Sperling visited his studio in 1621 and observed “a good number of young men each occupied on a different work, for which Rubens had provided a chalk drawing with touches of color added here and there.” Yet, Sperling added, “Kings and princes have heaped gifts and jewels upon him.”

In his mature years, he painted detailed models for large projects. These models stood alone as major works and along with draft sketches, hundreds of which have survived, give a glimpse into Rubens’ understanding of subjects, genius for composition and spontaneity of execution, the creative process itself. Among the painted models were designs made for a thriving tapestry market to which Rubens had ties through his family.

*The Gathering of the Manna*, on this month’s cover, was a painted model, part of a series, for tapestries commissioned by Archduchess Isabella of Spain, early employer and long-time patron. In this series, tapestries were shown within tapestries. *The Gathering of the Manna* was hung with cord and tassels from three lions’ heads attached to an architrave inside a framework of spiraling columns within an elaborate border.

The Old Testament story unfolded according to tradition. After leaving Egypt, the Israelites wandered in the desert for many days, starving. They appealed to God through Moses for food, “And in the morning … behold, upon the face of the wilderness there lay a small round thing, as small as the hoar frost on the ground.” The prophet, light emanating from his head, acknowledged the miracle, along with the figure on the right, possibly his brother Aaron. On the left, a woman with a child in tow held a basket filled with manna “like wafers made with honey.” Baskets high to collect the nourishment, the biblical figures engaged in a spirited dance, their gestures broad and theatrical, positions centered and balanced. They gathered and moved in perfect harmony. Moses lifted gaze and hand upward as in wonder and thanks. Mother and child headed gracefully toward the back.

Throughout history, as in biblical times, availability of food was a primary concern, one so great, it merited divine intervention. Safety of food also goes all the way back. The Israelites knew they were not to hoard manna for it would spoil and become poisonous. We have, for the most part, resolved availability. Although not optimally distributed, food is plentiful, and refrigeration and other means have eased spoilage. Yet, known foodborne pathogens and unspecified agents cause havoc in the United States and around the world, even if we have not been able to fully estimate the precise numbers of illnesses and deaths.

During a period of illness in 1623, English poet John Donne wrote about his disease and recovery, during which, like Moses in crisis, he “debated” with God. The book, Devotions upon Emergent Occasions, examined the stages of illness and commented on the human condition. “How little of man is the heart! And yet it is all by which he is; and this continually subject, not only to foreign poisons conveyed by others, but to intestine poisons, bred in ourselves by pestilential sicknesses.” These sicknesses, and others of a more sentimental nature, preoccupied Donne. In “Twicknam Garden” (1633), he wrote about love, a condition he likened to a spider because of its ability to infuse poison into everything, “The spider love, which transubstantiates all, / And can convert manna to gall.”

The poet’s graphic conversion of the sacred to the poisonous made for good reading, and the account of his illness was extremely popular in his day, even though he did not identify the illness, quantify, or provide any information that might help readers avoid pestilential sicknesses. For, then as now, reducing “intestine poisons” required information about the many factors along the farm-to-table pathway that can introduce or amplify microbial contamination and verify the feasibility and effectiveness of interventions. When it comes to food and illness caused by it, Donne’s biblical reference fits. The food by which we live can kill us and does, and unless we can choreograph the gathering and distribution of manna as well as Rubens did, the total picture with all its functioning and useful parts will continue to elude us.

**Figure:**
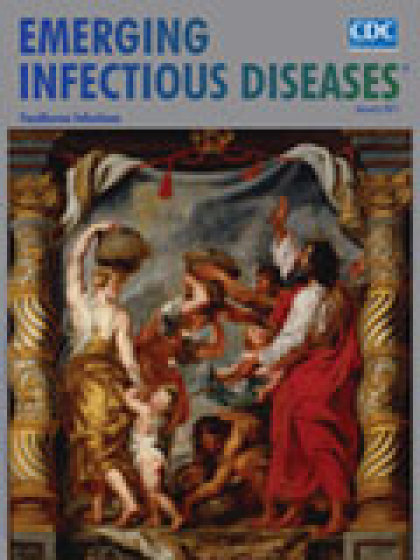
**Peter Paul Rubens (1577–1640) *The Gathering of the Manna* (c. 1625) Oil on canvas (487.68 cm × 411.48 cm)** Bequest of John Ringling, 1936, Collection of The John and Mable Ringling Museum of Art, the State Art Museum of Florida, a division of Florida State University
